# The impact of mentalization and psychological flexibility on college students’ mental health: A cross-sectional study

**DOI:** 10.1097/MD.0000000000048387

**Published:** 2026-04-17

**Authors:** Xuemei Li, Xiaosha Yang, Xinghua Wang

**Affiliations:** a Department of Psychiatry, The First Affiliated Hospital of Chongqing Medical University, Chongqing, China; b Department of Psychiatry, Hechuan District of Chongqing Mental Health Center, Chongqing, China.

**Keywords:** college students, mental health, mentalization, psychological flexibility

## Abstract

This study aimed to assess the mental health problems of college students and to explore whether mentalization and psychological flexibility are significantly associated with the mental health of college students. The mental health of 14,481 undergraduate students was evaluated using the Generalized Anxiety Disorder 7 and Patient Health Questionnaire-9. Mentalization was assessed using the Reflective Functioning Questionnaire, and psychological flexibility was evaluated using the Acceptance and Action Questionnaire-II and Cognitive Fusion Questionnaire. Mental health was measured using the Patient Health Questionnaire-9 and the 7-item Generalized Anxiety Disorder Scale. Multivariate and linear regression analyses were employed to examine the relationships among mentalization, psychological flexibility, and the mental health of college students. The Bootstrap procedure was used to explore the mediating role of psychological flexibility in mentalization and mental health. The mental health status of the college students revealed that mentalization and psychological flexibility were significantly related to anxiety and depression in college students. Age, gender, status of the only child, and marital status of parents were significantly related to anxiety and depression of college students. In addition, left-behind children were associated with depression, and higher hypermentalization scores were associated with higher anxiety and depression scores, indicating more severe anxiety and depression. Higher hypomentalization scores are associated with higher depression scores, indicating more severe depression. Psychological flexibility (cognitive fusion, acceptance, and action) partially mediated the relationship between hypermentalization and anxiety/depression scores, and a masking effect was observed in the relationship between hypomentalization and anxiety/depression scores. Anxiety and depression of college students are significantly related to mentalization and psychological flexibility.

## 1. Introduction

Mental health is a crucial indicator of overall well-being.^[1]^ The poor mental health of students in higher education has gradually become a public health and policy concern, with an increasing prevalence of mental health issues, including self-harm and suicide, among university students.^[2,3]^ Anxiety and depression have become widespread problems, with all universities reporting cases of students suffering from depression, and 99% of student surveys indicating severe anxiety symptoms.^[4]^ Some studies even suggest that university life itself can lead to changes in students’ mental health and that the mental health status of students varies across academic years.^[5]^ Stress is a key factor that influences mental health in psychological research. Academic stress is a common form of stress. High academic pressure can have a negative impact on the physical and mental health of students. A nationwide survey of university students in Norway revealed that over 21% of students had experienced suicidal thoughts at some point in their lives, and 7.2% reported having suicidal thoughts in the past year. The lifetime prevalence of nonsuicidal self-injury behaviors and thoughts was 19.6% and 22.6%, respectively, with approximately 4% of the students attempting suicide. These rates were higher among female, single, living alone, low-income, and immigrant students_._^[2]^

Mentalization capacity are mental processes that involve a person thinking about their own and other people’s thoughts, needs, feelings, wishes, and desires. Mentalization plays a crucial role in one’s mental health, social interactions, and emotional adjustment. It is closely related to personal personality development, emotional experience and expression ability, mental health, and so on.^[6–8]^ Psychological flexibility is a basic manifestation of health.^[9]^ Psychological flexibility refers to the ability of individuals to accept their own inner experience and take actions consistent with their own values when facing different mental processes. In acceptance and commitment therapy, low psychological flexibility is considered as the main source of suffering and its core goal is to improve psychological flexibility.^[10]^ Psychological flexibility can help improve students’ emotional regulation ability and enhance their learning success rate.^[11]^ In the medium- to long-term, psychological inflexibility can develop into a maladaptive process, becoming a risk factor for psychological difficulties and a decline in the quality of life.

From the above content, it can be seen that emotional state and mentalization, psychological flexibility are significantly correlated. However, at present, no study can show the correlation between mental health and mentalization and mental flexibility of college students. The nature of these interactions in relation to the mental health problems in this group remains unclear. This study aimed to assess the mental health problems of college students and to explore whether mentalization and psychological flexibility are significantly associated with the mental health of college students. Specifically, this study analyzed college students’ mental health in relation to academic stress, interpersonal challenges, emotional distress, and other relevant factors. It examines how mentalization – the ability to understand one’s own and others’ mental states – affects emotional regulation and behavioral choices.

Through systematic data collection and analysis, this study revealed a correlation between college students’ mental health, psychological flexibility, and mentalization, thereby providing scientific evidence and empirical insights for mental health interventions and support for students.

## 2. Methods

### 2.1. Participants and research design

Convenience sampling was conducted at a full-time university in Chongqing, China. An online survey was conducted and 14,722 questionnaires were collected from undergraduate university students. Among them, 231 questionnaires were not completed, resulting in at least 1 missing data. There were 7 cases with severely abnormal age data, and the age data were 0 to 2 years old. Finally, 14,481 valid data were obtained. All participants were clearly informed of the purpose of the study, the confidentiality measures for personal data, and the voluntary nature of participation before joining the study. Informed consent was obtained from all participants prior to their participation in the study.

### 2.2. Measures

#### 2.2.1. Demographics

General information included age, sex, educational level, place of household registration (urban, town, or remote rural areas), whether the individual was an only child, whether they had experienced being left behind, and their parents’ marital status.

#### 2.2.2. Mentalization

Mentalization was measured using the Reflective Functioning Questionnaire (RFQ), developed by Fonagy et al. The RFQ is a widely used tool for assessing RF and consists of 2 subscales: the Mental State Certainty Scale (RFQ-C, with items 1–6) and the Mental State Uncertainty Scale (RFQ-U, with items 2, 4, 5, 6, 7, and 8). Higher scores on these scales are associated with over-mentalization and low mentalization. The dual scoring mechanism, on the one hand, assesses the degree of hypomentalization and, on the other hand, assesses the degree of hypermentalization. As Items 2, 4, 5, and 6 appear simultaneously on both subscales, double scoring is required.^[12,13]^ In this study, Cronbach alpha coefficient for the full scale RFQ was 0.908. Cronbach alpha coefficient for the RFQ-C was 0.909. Cronbach alpha coefficient for the RFQ-U was 0.867.

#### 2.2.3. Psychological flexibility assessments

Psychological flexibility was measured using the Chinese versions of the Acceptance and Action Questionnaire-II (AAQ-II) and Cognitive Fusion Questionnaire (CFQ-F). The AAQ-II is used to assess experiential avoidance and consists of 7 self-report items on a 7-point scale, ranging from 1 = “Never” to 7 = “Always.” The total score ranges from 7 to 49, with higher scores indicating greater psychological rigidity.^[14–16]^ Cronbach alpha coefficient for the AAQ-II was 0.954.

The original questionnaire used in this study was the Chinese version of the CFQ-F developed by Gillanders et al. It consisted of 9 self-reported items rated on a 7-point Likert scale. The total score ranges from 9 to 63, with higher scores indicating greater psychological inflexibility.^[17–19]^ Cronbach alpha coefficient for the CFQ-F was 0.970.

#### 2.2.4. Mental health

Mental health was measured using the Patient Health Questionnaire-9 (PHQ-9) and the 7-item Generalized Anxiety Disorder Scale (GAD-7).

The PHQ-9 is a screening tool for depression and is widely used in clinical and research settings. It shows strong validity and reliability for detecting depression and evaluating its severity.^[20,21]^ The PHQ-9 is not only suitable for depression screening in various medical institutions, but also effective in the general population.^[22]^ The PHQ-9 consists of 9 self-report items rated on a 4-point scale to measure severity, with scores ranging from 0 (not at all) to 3 (nearly every day). The total score ranged from 0 to 27, with higher scores indicating more severe depression. In this study, the Cronbach alpha coefficient for the PHQ-9 was 0.905.

The GAD-7 scale shows strong validity and reliability for detecting generalized anxiety disorders and evaluating their severity.^[23,24]^ The GAD-7 is a self-reported anxiety questionnaire consisting of 7 items.^[25]^ This scale is a 4-point Likert scale. The total score obtained by adding up all the items ranges from 0 to 21. The higher the score, the more severe the anxiety. In this study, the Cronbach alpha coefficient for the GAD-7 was 0.928.

#### 2.2.5. Statistical analysis

Data analysis was conducted using the SPSSAU (Version 24.0 [Online Application Software], https://www.spssau.com). Descriptive statistics were used to examine research variables and their general characteristics. Univariate analysis was used to identify relationships between sociodemographic characteristics, psychological flexibility, mentalization, and mental health. Furthermore, to explore the correlation between the mental health status of college students and their psychological flexibility as well as their mentalization, a linear regression analysis was conducted. The Bootstrap procedure was used to investigate the mediating role of psychological flexibility in the relationship between mentalization and mental health. Statistical significance was set at a 2-tailed *P*-value of <.05.

#### 2.2.6. Ethical approval

This study was approved by the Ethics Committee of the First Affiliated Hospital of Chongqing Medical University (2024-121-01), in compliance with the ethical standards outlined in the Declaration of Helsinki. All participants provided informed consent, emphasizing their voluntary participation, anonymity, and confidentiality.

## 3. Results

A total of 14,481 valid questionnaires were collected for analysis. The age of the participants ranged from 16 to 32 years, with a mean age of 19.44 years (SD = 1.105). Males accounted for 73.2% of the total study population. The distribution of participants according to location was as follows: 42.7% from urban areas, 36.5% from towns, and 20.8% from rural areas. Among the respondents, 45.0% (n = 6513) were children, and the majority (70.7%, n = 10,244) did not have any left-behind experience. Most participants reported that their parents were married (85.8%, n = 12,423; Table 1).

**Table 1:** The study sample description (N = 14,481).

Sociodemographic	Total (N = 14,481)
Age (yr), mean (SD)	19.44 ± 1.105
Residence, n (%)	
Urban areas	6184 (42.7%)
Towns	5281 (36.5%)
Rural areas	3016 (20.8%)
Sex, n (%)	
Male	10,601 (73.2%)
Female	3880 (26.8%)
Being the only child or not, n (%)	
Yes	6513 (45.0%)
No	7968 (55.0%)
Experience of left behind, n (%)	
Yes	4237 (29.3%)
No	10,244 (70.7%)
Parental marital status, n (%)	
Married	12,423 (85.8%)
Divorced	1666 (11.5%)
Widowed	392 (2.7%)

According to the results of this study, 10,079 individuals (69.60%) did not exhibit symptoms of anxiety or depression, indicating that most participants had a normal emotional state. In contrast, 4402 individuals (30.40%) exhibited either anxiety or depression, with 2716 individuals (61.70% of those with anxiety or depression) presenting with both anxiety and depression symptoms (Table 2).

**Table 2:** Description of GAD-7 and PHQ-9 grades.

	PHQ-9 grading						
	None	Mild	Moderate	Moderately severe	Severe	Total	N%
GAD-7 grading							
None	10,079	779	64	10	2	10,934	75.5
Mild	793	1757	327	54	2	2933	20.3
Moderate	35	120	175	134	8	472	3.3
Severe	3	10	18	50	61	142	1.0
Total	10,910	2666	584	248	73	14,481	100
N%	75.3	18.4	4.0	1.7	0.5	100	

GAD-7 = Generalized Anxiety Disorder 7, PHQ-9 = Patient Health Questionnaire-9.

Table 3 shows that the RFQ-C, RFQ-U, acceptance and action, along with cognitive fusion, showed significant correlations with anxiety and depression scores (*P* < .001). Table 4 also indicates that age, gender, only-child status, and parental marital status are significantly correlated with anxiety (*P* < .01), as well as with depression (*P* < .001). In addition, left-behind experiences were associated with depression (*P* < .05).

**Table 3:** Factors affecting anxiety and depression scores (mean ± SD).

Sociodemographic	GAD-7	*T*/*F*/*R*	*P*	PHQ-9	*T*/*F*/*R*	*P*
Age (yr)		0.05	<.001‡		0.057	<.001‡
Sex		–9.341	<.001*		–4.858	<.001*
Male	2.29 ± 3.518			2.66 ± 4.086		
Female	2.91 ± 3.495			3.02 ± 3.865		
Residence, n (%)		0.339	.713†		1.509	.221†
Urban areas	2.49 ± 3.662			2.75 ± 4.156		
Towns	2.44 ± 3.424			2.71 ± 3.867		
Rural areas	2.44 ± 3.400			2.87 ± 4.052		
Being the only child or not, n (%)		–3.042	.002*		–3.71	<.001*
Yes	2.36 ± 3.533			2.62 ± 3.982		
No	2.54 ± 3.512			2.87 ± 4.086		
Experience of left behind, n (%)		1.3	.194*		2.558	.011*
Yes	2.52 ± 3.423			2.89 ± 3.998		
No	2.43 ± 3.563			2.70 ± 4.044		
Parental marital status, n (%)		13.555	<.001†		19.324	<.001†
Married	2.40 ± 3.456			2.68 ± 3.922		
Divorced	2.87 ± 3.952			3.33 ± 4.692		
Widowed	2.63 ± 3.559			2.87 ± 4.218		
RFQ-C		0.342	<.001‡		0.346	<.001‡
RFQ-U		–0.326	<.001‡		–0.324	<.001‡
CFQ-F cognitive fusion		0.467	<.001‡		0.454	<.001‡
AAQ-II acceptance and action		0.501	<.001‡		0.496	<.001‡

AAQ-II = Acceptance and Action Questionnaire-II, CFQ-F = Cognitive Fusion Questionnaire, GAD-7 = Generalized Anxiety Disorder 7, PHQ-9 = Patient Health Questionnaire-9, RFQ-C = Certainty of Mental States Scale, RFQ-U = Uncertainty of Mental States Scale.

* *t*-value.

† *F*-value.

‡ *R*-value.

**Table 4:** Linear regression of mentalization, psychological flexibility, and anxiety-depressive symptoms.

Independent variable	GAD-7			PHQ-9		
	*B*	95% CI		*B*	95% CI	
		Lower	Upper		Lower	Upper
	–2.944			–2.845		
RFQ-C	0.875	0.744	1.005	1.117	0.967	1.266
RFQ-U	0.511	0.436	0.585	0.529	0.443	0.615
CFQ-F cognitive fusion	0.050	0.044	0.057	0.047	0.040	0.055
AAQ-II acceptance and action	0.151	0.142	0.160	0.176	0.165	0.187

Age, gender, only-child status, left-behind experience, and parental marital status were controlled.

AAQ-II = Acceptance and Action Questionnaire-II, CFQ-F = Cognitive Fusion Questionnaire, GAD-7 = Generalized Anxiety Disorder 7, PHQ-9 = Patient Health Questionnaire- 9, RFQ-C = Certainty of Mental States Scale, RFQ-U = Uncertainty of Mental States Scale.

Linear regression analysis further revealed that RFQ-C was significantly associated with anxiety (*B* = 0.875, β = 0.117, *P* < .001) and depression (*B* = 1.117, β = 0.131, *P* < .001); RFQ-U was significantly associated with anxiety (*B* = 0.511, β = 0.148, *P* < .001) and depression (*B* = 0.529, β = 0.134, *P* < .001); cognitive fusion was significantly associated with anxiety (*B* = 0.050, β = 0.199, *P* < .001) and depression (*B* = 0.047, β = 0.164, *P* < .001); acceptance and action were significantly associated with anxiety (*B* = 0.151, β = 0.382, *P* < .001) and depression (*B* = 0.176, β = 0.389, *P* < .001). The analysis (Table 4) controlled for age, gender, whether the person was an only child, history of being left behind, and parents’ marital status.

To further explore the relationship between college students’ anxiety and depression and mentalization, as well as psychological flexibility, this study further investigates whether psychological flexibility acts as a mediator between mentalization and anxiety and depression. First, the predictor variables were standardized using anxiety-depressive emotional scores as the dependent variables. The 2 dimensions of mentalization were selected as predictor variables, while AQQ-II and CFQ-F scores were chosen as mediating variables for mediation effect testing. The number of bootstrap resamplings was set at 5000.

Psychological flexibility partially mediated the relationship between RFQ-C and scores of anxiety and depression. With anxiety scores as the dependent variable, the mediation effect was 0.874 (cognitive fusion: 0.038; acceptance and action: 0.484). With depression scores as the dependent variable, the mediation effect was 0.924 (cognitive fusion: 0.358; acceptance and action: 0.565).

Psychological flexibility masked the effect of RFQ-C on anxiety and depression scores. With anxiety as the dependent variable, the mediation effect was −1.182 (cognitive fusion: −0.414; acceptance and action: −0.768). With depression as the dependent variable, the mediation effect was −1.288 (cognitive fusion: −0.391; acceptance and action: −0.897; Table 5).

**Table 5:** The mediating role of psychological flexibility between mentalization and anxiety-depression emotions.

	GAD-7				PHQ-9			
	Value	95% CI		Relative effect proportion%	Value	95% CI		Relative effect proportion%
		Lower	Upper			Lower	Upper	
RFQ-C								
Total effect	1.737	1.603	1.872	100	2.041	1.888	2.195	100
Direct effect	0.874	0.743	1.004	50.3	1.117	0.967	1.267	54.7
Indirect effect	0.864	0.106	0.126	49.7	0.924	0.098	0.118	45.3
CFQ-F cognitive fusion	0.38	0.043	0.058	21.85	0.358	0.034	0.05	17.56
AAQ-II acceptance and action	0.484	0.057	0.073	27.86	0.565	0.058	0.075	27.69
RFQ-U								
Total effect	–0.672	–0.734	–0.609	100	–0.759	–0.83	–0.688	100
Direct effect	0.51	0.436	0.585	–	0.529	0.443	0.615	–
Indirect effect	–1.182	–0.358	–0.326	–	–1.288	–0.341	–0.309	–
CFQ-F cognitive fusion	–0.414	–0.137	–0.102	81.07	–0.391	–0.116	–0.08	73.86
AAQ-II acceptance and action	–0.768	–0.238	–0.207	150.56	–0.897	–0.243	–0.21	169.64

The masking effect ratio refers to the proportion of the mediation effect relative to the direct effect.

AAQ-II = Acceptance and Action Questionnaire-II, CFQ-F = Cognitive Fusion Questionnaire, GAD-7 = Generalized Anxiety Disorder 7, PHQ-9 = Patient Health Questionnaire- 9, RFQ-C = Certainty of Mental States Scale, RFQ-U = Uncertainty of Mental States Scale.

## 4. Discussion

To the best of our knowledge, this is the first study to use a sample of university students to assess the relationship between psychological flexibility, mentalization, and mental health. This study is the first to examine the link between university students’ mentalization and their anxiety-depressive symptoms, as well as to explore how psychological flexibility mediates the relationship between mentalization and mental health. The PHQ-9 and GAD-7 rank among the world’s most recognized mental health screening tools, showing strong consistency across varied populations and suitability for mental health screening.^[26]^ In actual use, the PHQ-9 and GAD-7 are employed across multiple areas, including general healthcare, mental health nursing, and the screening and evaluation of diverse population groups (e.g., university students and chronically ill patients).^[27]^ These tools are user-friendly and can efficiently identify individuals needing further psychological assessment and support.

Mental health issues among university students are a significant concern in many countries, around the world. A meta-analysis of 64 studies with a total of 100,187 participants revealed that the comorbid prevalence rates of depression and anxiety symptoms among university students were 33.6% (95% CI, 29.3%–37.8%) and 39.0% (95% CI, 34.6%–43.4%), respectively. The analysis also indicated that the highest prevalence of depression was found in African regions, low- and middle-income countries, and medical school students.^[28]^

The study shows that among college students, 10,934 (75.5%) reported no anxiety, 20.3% had mild anxiety, 3.3% had moderate anxiety, and 1.0% had severe anxiety. Regarding depression, 10,910 students (75.3%) reported no depression, 18.4% had mild depression, 4.0% had moderate depression, 1.7% had moderate-to-severe depression, and 0.5% had severe depression. Our study found that 4402 students (30.40% of the total sample) experienced either anxiety or depression, highlighting that emotional issues are widespread among college students. Furthermore, among those with emotional problems, 2716 (61.70%) had both anxiety and depression, suggesting a high comorbidity rate of anxiety and depression among students experiencing emotional difficulties.

Multiple studies indicate that women experience higher rates of anxiety and depression compared to men,^[29]^ and this gender difference is commonly observed across various age groups and social contexts. In the present study, sex was found to be a significant factor affecting both the anxiety and depression scores. Some studies have suggested a correlation between residence and anxiety-depression symptoms. For instance, Amoran et al.^[30]^ The report indicates that whether teenagers live in rural areas or urban areas has no impact on their rates of anxiety disorders and depression. In the current study, no significant differences were found between residence and anxiety-depression scores. A longitudinal study examining the mental health of Chinese university students found that those with left-behind backgrounds exhibited more psychotic symptoms, such as depression, anxiety, somatic disorders, obsessive-compulsive symptoms, suicide attempts, and intentional self-harm.^[31]^ In the research conducted in this article, it was found that “left-behind experience” had a significant impact on the depression score, but had no effect on the anxiety score. Parental divorce is one of the most stressful events in adolescents’ lives and is often associated with long-term emotional and behavioral problems.^[32]^ This study reveals that the marital status of parents has a significant impact on the anxiety and depression levels of college students.

Based on the above content, this study found significant differences in anxiety and depression scores related to gender and parental marital status, but no significant differences based on place of residence. The experience of being left behind is associated with differences in depression scores but not in anxiety scores.

In recent years, the mental health issues of university students have received increasing attention. The present study reports the preliminary results of the first phase of the World Health Organization World Mental Health International College Student Project. The present study reports initial results from the first stage of the World Health Organization World Mental Health International College Student Project, in which a series of surveys in 19 colleges across 8 countries (Australia, Belgium, Germany, Mexico, Northern Ireland, South Africa, Spain, and the United States) were carried out to estimate the prevalence and basic sociodemographic correlates of common mental disorders among first-year college students; web-based self-report questionnaires administered to incoming first-year students (45.5% pooled response rate) screened for 6 common lifetime and 12-month DSM-IV mental disorders, including major depression, mania/hypomania, generalized anxiety disorder, panic disorder, alcohol use disorder, and substance use disorder. We focused on 13,984 full-time students: 35% screened positive for at least 1 common lifetime disorder, and 31% screened positive for at least one 12-month disorder.^[33]^

The study of college students’ inherent mentalization capabilities pertains both to mentalization-based treatment and their psychological well-being. This study employed the RFQ to evaluate 2 aspects of mentalization among college students: low agreement reflected hypermentalizing on the RFQ-C scale, and high agreement reflected genuine mentalizing (acknowledging the opacity of mental states). On the RFQ-U scale, very high scores reflected a stance characterized by an almost complete lack of knowledge about mental states, whereas lower scores reflected acknowledgment of the opaqueness of one’s own mental state and that of others, a characteristic of genuine mentalization, and were positively correlated with anxiety and depression scores. The RFQ-U scale score was negatively correlated with anxiety and depression scores (Table 3). The scores of anxiety and depression are positively correlated with acceptance and action, as well as cognitive integration (Table 3). This suggests that mentalization ability and psychological flexibility were significantly correlated with anxiety and depressive symptoms (*P* < .001).

Moreover, based on these findings, mentalization and psychological flexibility might not only correlate directly with anxiety and depression scores but also exert an indirect influence. Therefore, a bootstrap method was used to test the mediating effect of psychological flexibility on the link between mentalization and anxiety-depression scores. The results showed that psychological flexibility played a partial mediating role in the relationship between the RFQ-C and anxiety-depression scores, whereas psychological flexibility had a masking effect on the relationship between the RFQ-U and anxiety-depression mood scores (Table 5). In the mediation analysis of this study, there might be a specific relationship between psychological flexibility and RFQ-U, or between psychological flexibility and anxiety and depression scores, thereby exerting a masking effect. This further indicates that psychological flexibility may play a complex role in the mediation process. These results indicate a strong connection between psychological flexibility, mentalization, and mental well-being, implying that enhancing psychological flexibility and mentalization could aid in recovering from psychological issues.

Finally, this study had certain limitations. In this study, when assessing mentalization, only the “the Mentalization RFQ scale” was used; while in measuring psychological flexibility, only the CFQ-F and the AAQ-II were employed. Future research should use more multidimensional measurement tools to reflect participants’ psychological flexibility and mentalization abilities more comprehensively. Second, in this study, all the data used were from the same university; therefore, our findings may better reflect the mentalization, psychological flexibility, and mental health of university students in 1 specific region. Lastly, this study is cross-sectional and, thus, cannot establish causal relationships between the variables. Future research could conduct longitudinal studies to verify the relationships between mentalization, psychological flexibility, and mental health and explore the mediating role of psychological flexibility.

## 5. Conclusion

In this study, the effects of over-mentalization and low mentalizing among college students on and depression were opposite. Over-mentalization was positively correlated with anxiety and depression, while low mentalizing had the opposite effect. Psychological flexibility (cognitive fusion, acceptance, and action) played a partial mediating role in both over-mentalization, anxiety, and depression scores, and there was a masking effect on the relationship between low mentalization, anxiety, and depression scores.

## Author contributions

**Conceptualization:** Xinghua Wang.

**Methodology:** Xiaosha Yang.

**Formal analysis:** Xiaosha Yang.

**Data curation:** Xinghua Wang, Xiaosha Yang.

**Investigation:** Xiaosha Yang.

**Project administration:** Xuemei Li.

**Resources:** Xuemei Li.

**Software:** Xuemei Li.

**Visualization:** Xuemei Li.

**Supervision:** Xinghua Wang.

**Validation:** Xinghua Wang.

**Writing – original draft:** Xuemei Li.

**Writing – review & editing:** Xinghua Wang.
